# A Bibliometric Analysis of the Landscape of Parathyroid Carcinoma Research Based on the PubMed (2000–2021)

**DOI:** 10.3389/fonc.2022.824201

**Published:** 2022-02-07

**Authors:** Chenzhe Feng, Chuwen Tian, Leyi Huang, Haolin Chen, Yeqian Feng, Shi Chang

**Affiliations:** ^1^ Department of General Surgery, Xiangya Hospital Central South University, Changsha, China; ^2^ Xiangya School of Medicine, Central South University, Changsha, China; ^3^ Department of Mathematics, University of California (UC) Davis, Davis, CA, United States; ^4^ Department of Oncology, Second Xiangya Hospital of Central South University, Changsha, China; ^5^ Clinical Research Center For Thyroid Disease In Hunan Province, Changsha, China; ^6^ Hunan Provincial Engineering Research Center for Thyroid and Related Diseases Treatment Technology, Changsha, China

**Keywords:** parathyroid carcinoma, machine learning, natural language processing, publication analysis, PubMed

## Abstract

**Introduction:**

The purpose of this study was to assess the landscape of parathyroid carcinoma research during the last 22 years using machine learning and text analysis.

**Method:**

In November 2021, we obtained from PubMed all works indexed under the mesh subject line “parathyroid carcinoma”. The entire set of search results was retrieved in XML format, and metadata such as title, abstract, keywords, mesh words, and year of publication were extracted for bibliometric evaluation from the original XML files. To increase the specificity of the investigation, the Latent Dirichlet allocation (LDA) topic modeling method was applied.

**Results:**

The paper analyzed 3578 papers. The volume of literature related to parathyroid cancer has been relatively flat over the past 22 years. In the field of parathyroid cancer research, the most important topic of clinical interest is the differential diagnosis. Ultrasound and MIBI are the most commonly used imaging methods for localization. In terms of basic research, the mechanisms of gene mutation and local tumor recurrence are the focus of interest.

**Conclusion:**

There are huge unmet research needs for parathyroid carcinoma. Improving the diagnosis rates of parathyroid cancer by clinicians and establishing new and reliable molecular pathological markers and new image localization techniques will continue to be the focus of future research.

## Introduction

Parathyroid carcinoma is a rare endocrine malignancy (1). The exact cause of parathyroid cancer is still unclear, and the clinical manifestations are diverse and difficult to diagnose differently from many other diseases. Parathyroid carcinoma is characterized by high serum ionized calcium and parathyroid hormone levels, but the lack of specific clinical, biochemical, or radiological features makes it difficult to distinguish it from the more common adenomas or parathyroid hyperplasia (2). Parathyroid cancer is often underdiagnosed due to its small size and inadequate clinical recognition, and most patients die from uncontrolled severe hypercalcemia rather than the tumor itself (3). Complete surgical excision is the only known possible cure for PC. Therefore, it is extremely important to improve the level of clinicians in recognizing and diagnosing parathyroid cancer, to make a clear diagnosis as early as possible, and to select the most suitable surgical procedure for timely treatment to improve the survival rate and prolong the survival time of patients.

Bibliometrics refers to the application of mathematical and statistical methods to calculate and analyze textual data, usually to demonstrate the nature and trends of the development of a discipline (4, 5). Presenting the current state of research across the field will help us understand the current landscape of parathyroid cancer, analyze research priorities and gaps, and better guide the future direction of research on this rare disease.

In this paper, we choose a natural language processing approach and apply artificial intelligence to study the topic of literature. The aim is to identify discrete topics in a large number of unstructured texts based on the PubMed database and explore the topics through data analysis and artificial intelligence to present the current status of research in the field of parathyroid cancer, to focus on the changing direction of research, to present the problems of current research, and to try to guide the areas worthy of further exploration. In addition, these results may help inform funding agencies about areas with the greatest research opportunities.

## Method

This study continues to follow previous research ideas and methods (4, 5). We downloaded all publications indexed under the mesh subject line “parathyroid carcinoma” from the public version of PubMed in November 2021. The full records of the search results were downloaded in XML format, and we extracted metadata from them. The extracted metadata included the title, abstract, keywords, year of publication, and mesh term of each article.

We use the most classical Latent Dirichlet Allocation (LDA) topic modeling method for literature topic modeling to accurately identify the topic of each study. LDA is an unsupervised machine learning technique that can be used to identify latent topic information in large-scale document collections or corpora. This approach treats each document as a word frequency vector, thus transforming textual information into numerical information that can be easily modeled. In the three-level structure of words, topics, and documents, each document represents a probability distribution composed of some topics, and each topic represents a probability distribution composed of many words. Thus, the LDA algorithm gives the probability that an article examines a particular research topic based on the frequency of feature words in each document (6, 7).

We set the number of identified topics to 50. Based on the analysis of the article abstracts, we defined the topic with the highest probability of belonging to each article calculated by LDA as the main topic of each article. We named the topics based on manual checking of abstract terms and MeSH words regarding article abstracts. We used the Louvain algorithm for cluster analysis to construct a network by the similarity between different topics and identify common communities of related topics based on the relationships between topics. For each article, we identify the two topics with the highest probability of their attribution. Links between topics are established based on the frequency of each topic’s occurrence in each document.

The relevant Python and R language code can be found in the cited literature (4, 5). All descriptive statistics are reported as the mean ± standard deviation. The network visualization in the article was performed with Excel and Gephi (https://gephi.org/) (8, 9). This article is a bibliometric analysis and therefore does not require approval from an institutional review board or ethics committee.

## Results

**Figure 1:**
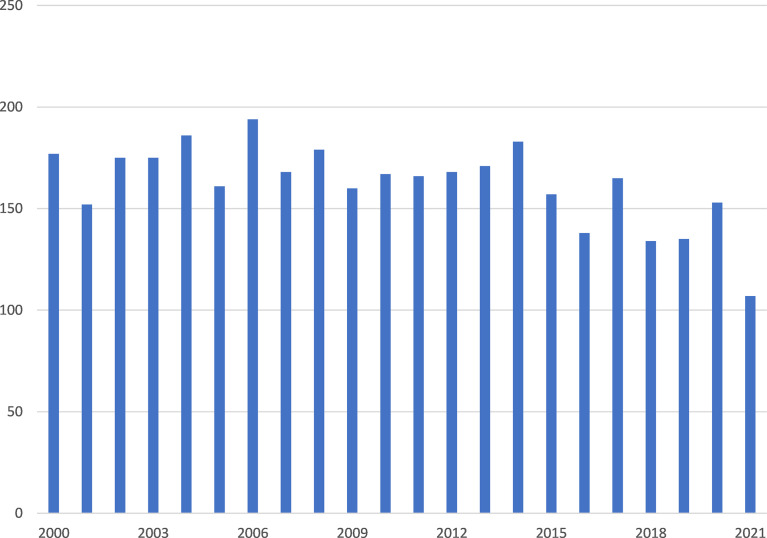
PubMed search results: articles per year.

In total, we identified 3578 publications, including 3315 journal articles, 1529 case reports, and 470 reviews. The number of publications per year from 2000 to 2021 does not show a clear trend (**Figure 1**) and even shows signs of a slow decrease since 2015.

### MeSH Analysis

**Figure 2:**
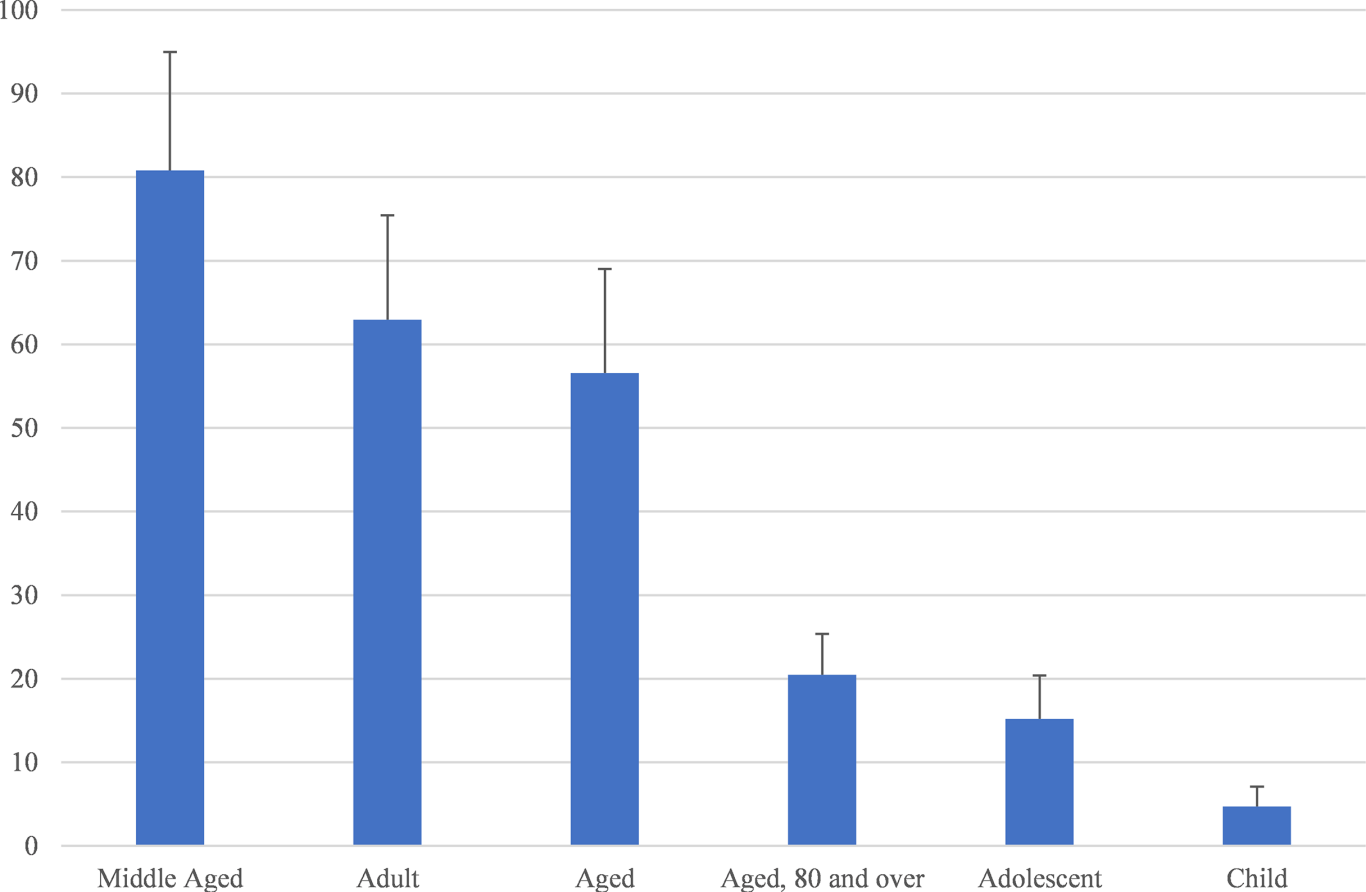
Annual output of literature, broken down by age group.

**Table 1** shows the top 20 mesh terms that appeared in the retrieved articles that were thematically related. The most frequently occurring terms include A*denoma, Parathyroidectomy, Treatment Outcome, Primary Hyperparathyroidism, Hyperparathyroidism, Technetium Tc 99m Sestamibi*, and *Radionuclide Imaging*. **Figure 2** illustrates the percentage of the number of relevant research publications for different age groups over the last 22 years. The age groups we identified were *child, adolescent, adult, middle-aged, aged*, and *aged over 80*. Articles dealing with more than one age group were included in the total number of publications for all corresponding age groups. It was found that the *middle-aged* and *adult* groups accounted for more publications related to parathyroid cancer, followed by the *old* group.

**Table 1:** Overall ranking of research foci in the past 22 years.

Rank	MeSH term	Record of occurrence in publications
**1**	Adenoma	2233
**2**	Parathyroidectomy	1166
**3**	Hyperparathyroidism, Primary	999
**4**	Hyperparathyroidism	970
**5**	Technetium Tc 99 m Sestamibi	655
**6**	Radionuclide Imaging	526
**7**	Radiopharmaceuticals	509
**8**	Hypercalcemia	485
**9**	Treatment Outcome	457
**10**	Diagnosis, Differential	428
**11**	Ultrasonography	361
**12**	Tomography, X-ray Computed	342
**13**	Sensitivity and Specificity	288
**14**	Minimally Invasive Surgical Procedures	224
**15**	Tomography, Emission-Computed, Single-Photon	224
**16**	Hyperplasia	214
**17**	Preoperative Care	164
**18**	Choristoma	163
**19**	Multiple Endocrine Neoplasia Type 1	159
**20**	Immunohistochemistry	156

**Figure 3:**
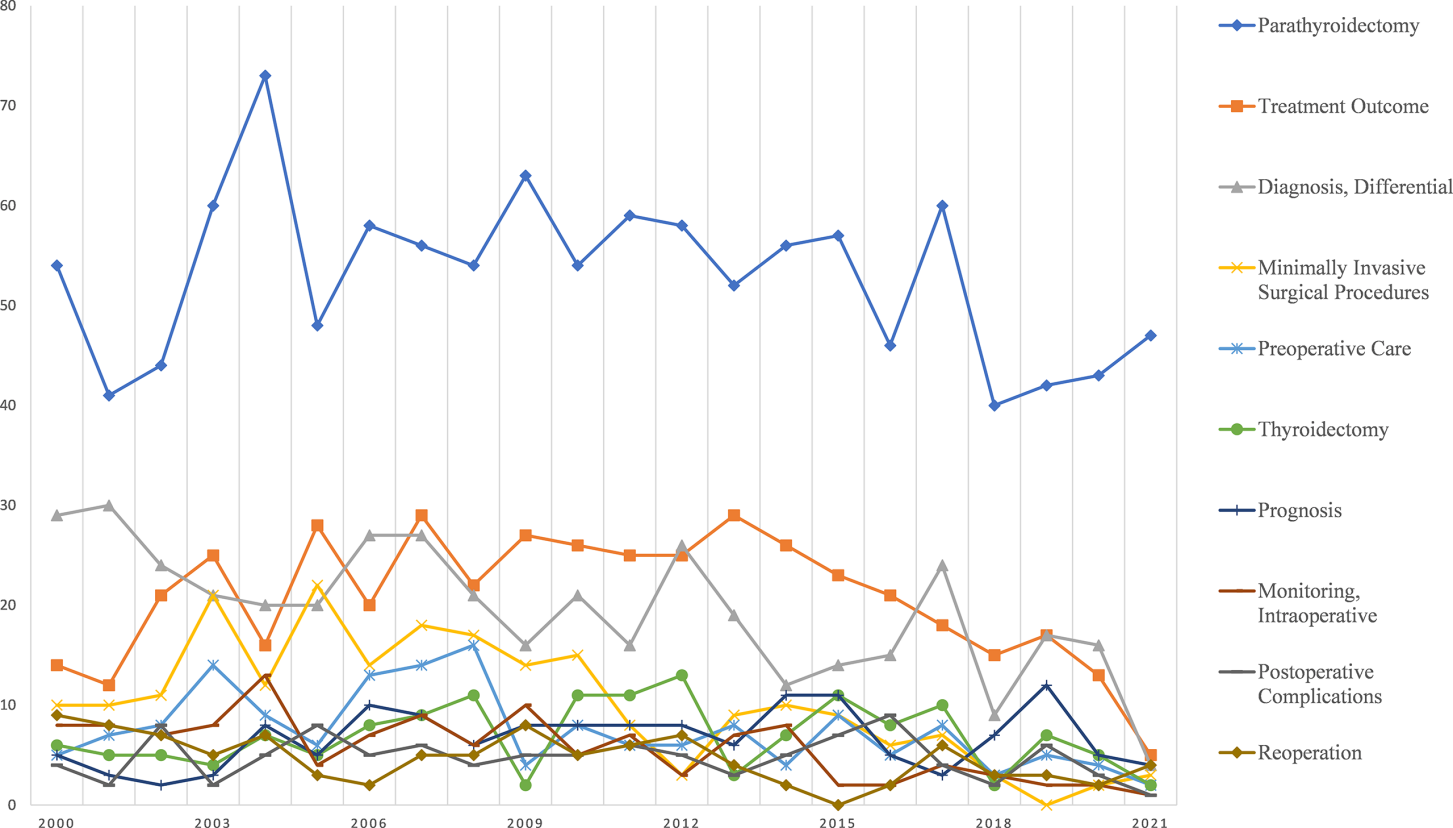
Research foci trends related to treatment.

**Figure 4:**
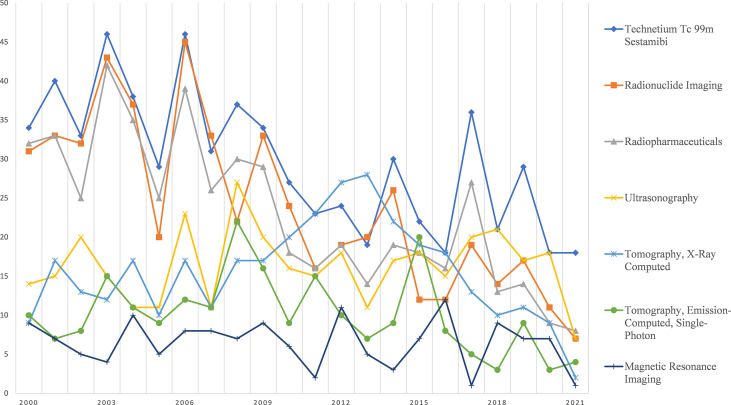
Research focus trends related to diagnostic tools.

**Figure 3** shows the trend of the 10 most popular topics related to the diagnosis and treatment of parathyroid cancer. The number of publications related to each topic has not fluctuated significantly in the last 21 years. Among them, *Parathyroidectomy* has been receiving much more attention than other topics such as *Treatment Outcome*, *Differential Diagnosis* for many years. **Figure 4** shows the trend of the 7 most popular topics in terms of imaging methods for parathyroid cancer. Among these seven most popular examination methods, *Technetium Tc 99 m Sestamibi*, *Radionuclide Imaging*, and *Radiopharmaceuticals* were more popular until 2006, after which they started to decline.

**Figure 5:**
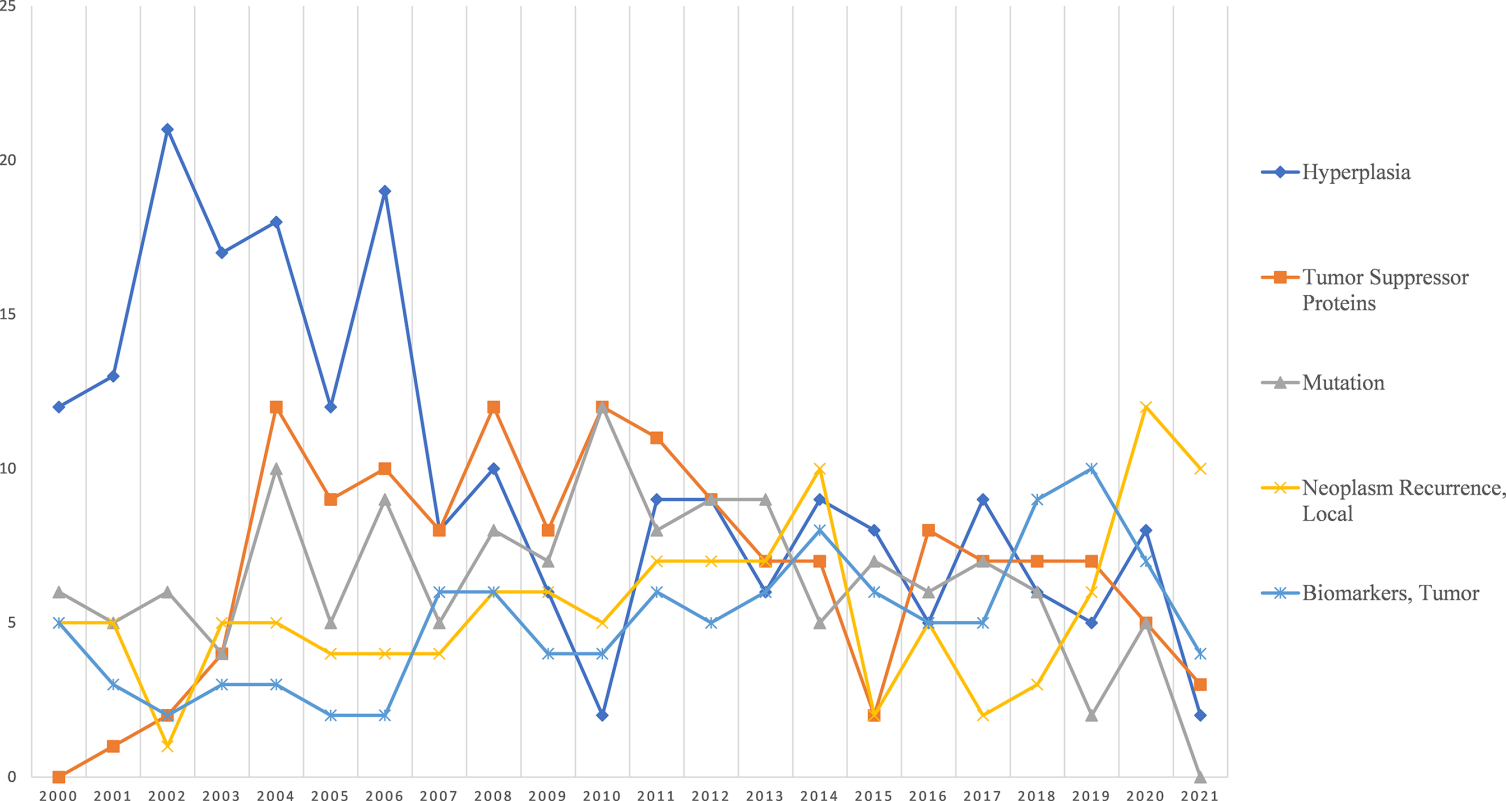
Research focus trends related to basic research.

**Figure 5** shows the trend of five hot topics of basic research related to parathyroid cancer. Basic research on parathyroid cancer is mainly focused on *Hyperplasia*, *Tumor Suppressor Proteins*, *Mutation*, *Local Neoplasm Recurrence*, and *Tumor Biomarkers*. Among them, *Hyperplasia* has received more attention in the early years and has shown a decreasing trend since 2006.

### Latent Dirichlet Allocation

**Figure 6:**
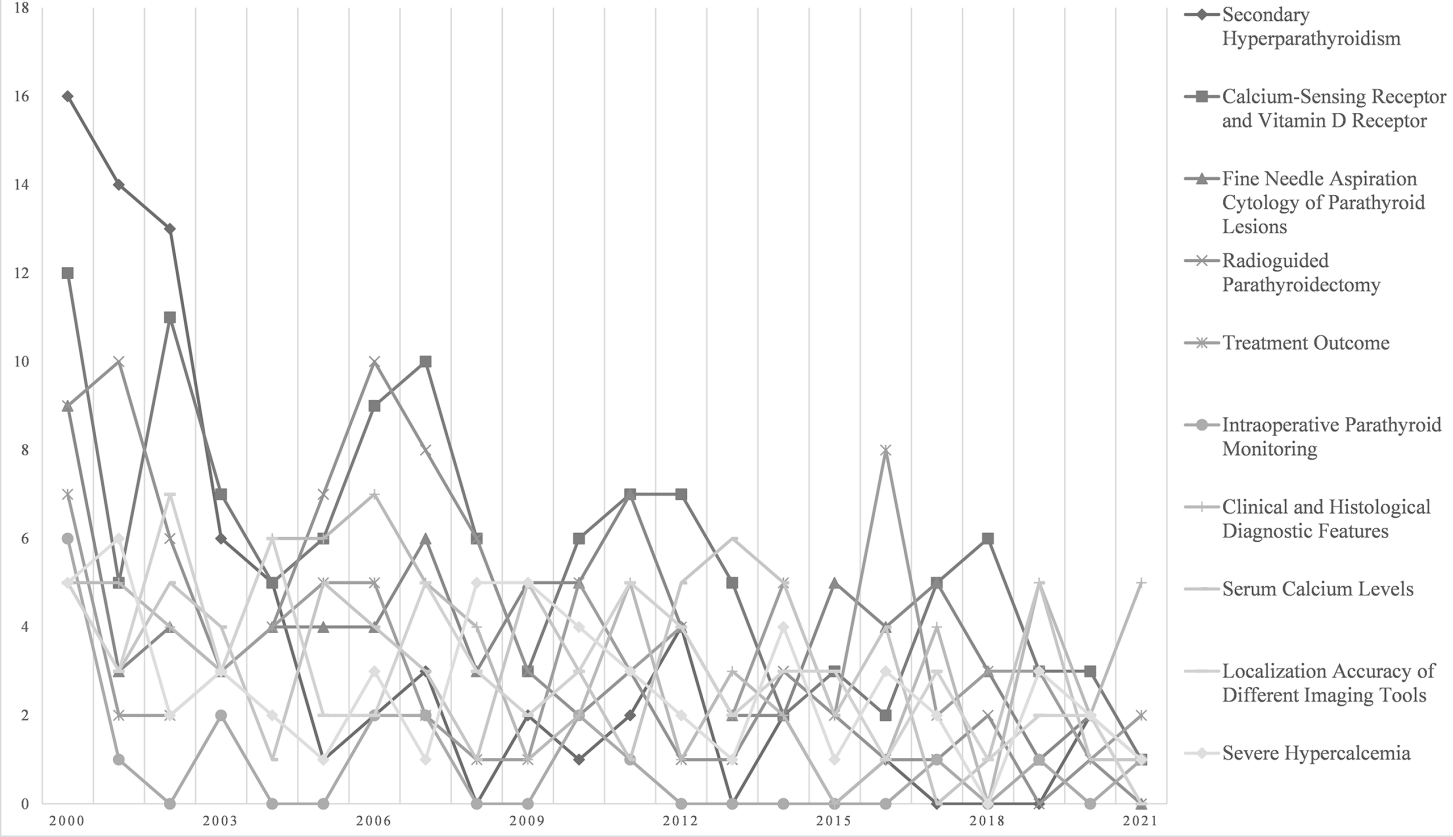
Latent Dirichlet allocation (LDA) analysis: increase in topic area articles per year.

LDA themes derived from publication abstracts show more details of high-frequency research themes in the literature. **Figure 6** shows the top 10 LDA topics with the greatest change in volume over the past 22 years. *Secondary Hyperparathyroidism*, *Calcium-Sensing Receptor, Vitamin D Receptor*, and *Fine Needle Aspiration Cytology of Parathyroid Lesions* are in the top three. Most of the topics were studied more in the early years but overall showed a decline year by year.

**Figure 7:**
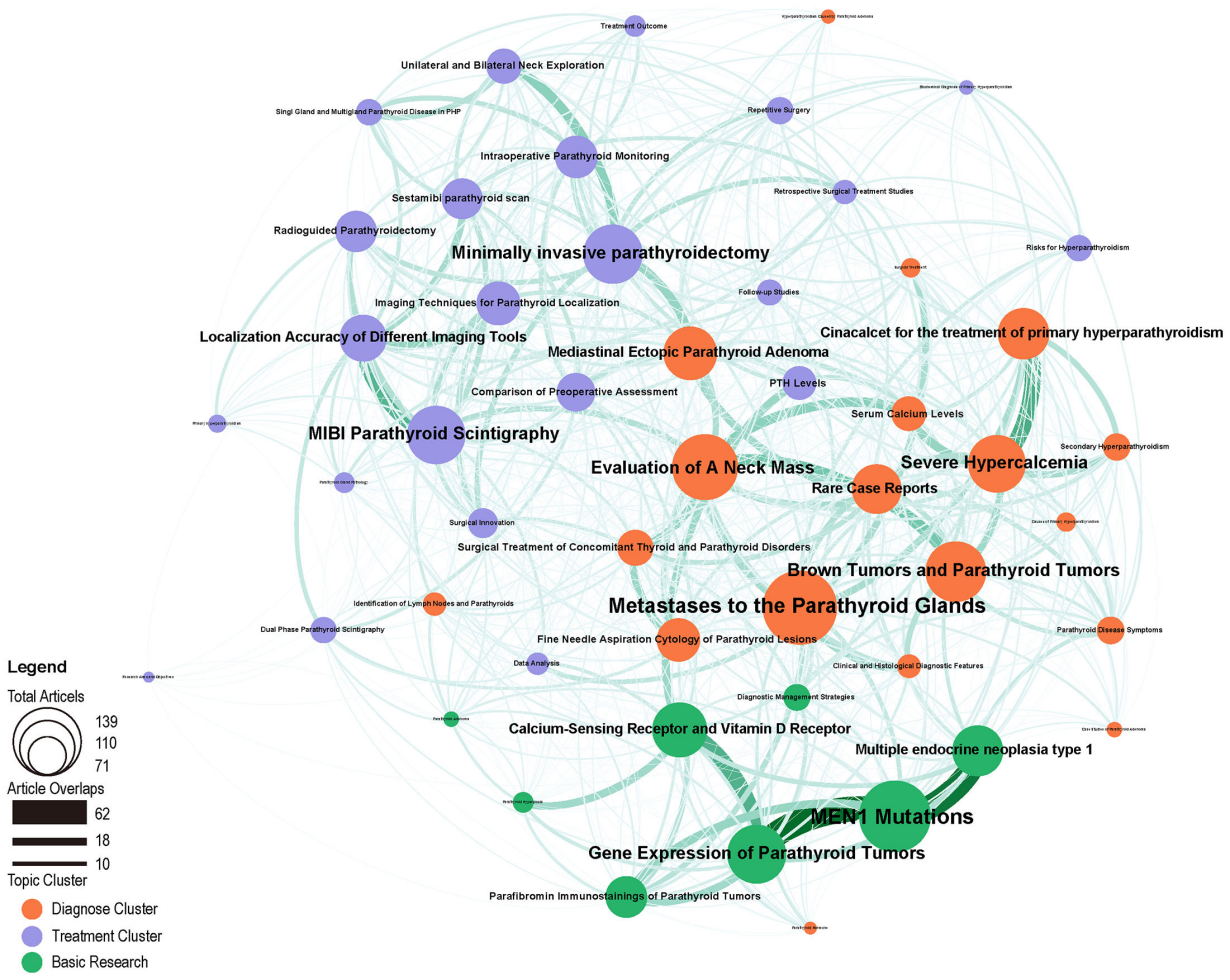
LDA research topic cluster network: inter-and intrarelationships. For each topic, the size of the bubbles represents the number of articles associated with it. The lines between the bubbles indicate that the two topics contain articles that use common terminology, and their thickness represents the size of the relationship between the topics. Different colors represent different clusters, orange for diagnosis, purple for treatment, and green for basic research.

Our network analysis of research themes presents areas where clusters of themes with high similarity co-occur and show the connections and strength between different themes (**Figure 7**). Three thematic network clusters were identified by the Louvain method, with strong intracluster relationships between publications within each thematic network cluster. They can be grouped into three directions in the field of parathyroid cancer research: diagnosis, treatment, and basic research. For each theme, the size of the bubbles represents the number of papers associated with it. The lines between the bubbles indicate that the two themes contain articles that use common terminology, and their thickness represents the magnitude of the relationship between the themes.

In the diagnosis cluster, the most studied topics were Rare Case Reports, Evaluation of A Neck Mass, Fine Needle Aspiration Cytology of Parathyroid Lesions. Among them, Rare Case Reports, Evaluation of A Neck Mass, Brown Tumors, and Parathyroid Tumors showed a strong correlation. Severe Hypercalcemia was closely associated with Cinacalcet for the treatment of primary hyperparathyroidism and Serum Calcium Levels. There was also a strong association between Serum Calcium Levels and Evaluation of A Neck Mass. Unilateral and Bilateral Neck Exploration and Minimally invasive parathyroidectomy were both strongly associated with Intraoperative Parathyroid Monitoring.

In the Treatment cluster, the most studied topics are Follow-up Studies, Comparison of Preoperative Assessment, MIBI Parathyroid Scintigraphy, and Retrospective Surgical Treatment Studies. It is worth noting that MIBI Parathyroid Scintigraphy has a strong correlation with Localization Accuracy of Different Imaging Tools.

In the Basic Research cluster, its hotspots focus on Multiple endocrine neoplasia type 1, Parafibromin Immunostainings of Parathyroid Tumors, MEN1 Mutations and Calcium-Sensing Receptor and Vitamin D Receptor. Within clusters, Multiple endocrine neoplasia type 1, Gene Expression of Parathyroid Tumors was strongly correlated with MEN1 Mutations. A significant association between Minimally invasive parathyroidectomy and Mediastinal Ectopic Parathyroid Adenoma was observed. Evaluation of A Neck Mass was significantly associated with PTH levels.

## Discussion

The number of publications on parathyroid cancer has remained relatively flat over the past 22 years, in contrast to the previous dramatic increase in the number of publications on similar approaches in other fields (4, 5). Although parathyroid cancer is a rare disease that may not be studied in the same pattern as any of the common diseases, the rarity does not simply explain why we have not seen more literature. Of particular note, different studies have suggested that parathyroid cancer may have been underdiagnosed. Lee et al. studied patients diagnosed with parathyroid cancer from 1988 to 2003 in the U.S. Surveillance, Epidemiology, and End Results (SEER) cancer registry data. They found a 60% increase in the incidence of parathyroid cancer during this 16-year study period (10). Kong et al. studied the Korean National Health Insurance Service database from 2002-2017 in Korea. They found that in this national cohort in Korea, the prevalence of PC increased over time, from 3.8 in 2003 to 6.6/10,000,000 person-years in 2016 (11). Several studies suggest an increase in the incidence of parathyroid cancer (12, 13).

Our study identifies that differential diagnosis is a current research priority in the clinical aspects of parathyroid cancer. A definitive diagnosis is important because, in parathyroid cancer, the only possibility of cure is complete surgical excision, preferably at the time of the first operation (2, 14). Currently, the diagnosis of parathyroid cancer is mostly made through postoperative pathology (15). Difficulties in timely recognition mean that a proportion of patients undergo partial resection rather than the gold standard of total resection. This was demonstrated in a previous series of case reports where a simple local excision was performed most often at the time of surgery (1). Even with postoperative pathology, the pathologic diagnosis and differential diagnosis of parathyroid adenoma (PA) remains difficult (16). Therefore, the differential diagnosis of parathyroid cancer will be one of the major research priorities in the field of parathyroid cancer.

In addition to diagnosis, the treatment of parathyroid cancer is equally challenging. Radical surgery is by far the most important treatment, with overall good survival rates at five and ten years (17). Early diagnosis and treatment are important factors in the prognosis of patients (18). Intraoperative parathyroid hormone (PTH) monitoring (ioPTH) can be a reliable marker for predicting malignancy in parathyroidectomy. In particular, in minimally invasive parathyroidectomy, ioPTH can avoid bilateral neck exploration and ensure successful surgical excision of almost all hyperfunctional tissue (19). At the same time, the use of ioPTH improves the sensitivity of detecting polyadenopathy and minimizes the need and risk associated with recurrent surgery (19). The patient was re-explored during ipsilateral hemithyroidectomy to reduce the rate of local recurrence. In addition, prophylactic lymphadenectomy is not necessary (20).

This study further provides further analysis on imaging-related topics. Our study found that ultrasound (US) and MIBI (99mtc-sec scan ratio imaging) imaging have been the most studied subjects in the past, and indeed, both are the most commonly used imaging studies to detect parathyroid abnormalities in patients with PHPT. However, the intensity of MIBI uptake does not necessarily distinguish between malignant and benign parathyroid lesions. Ultrasound US plays an important role in the preoperative localization of enlarged parathyroid glands but is less specific in distinguishing PC from adenoma or hyperplasia (21). The study of novel image localization techniques in parathyroid cancer will be one of the research priorities.

A major challenge for parathyroid cancer is to distinguish between PC and atypical parathyroid adenoma. To date, there is a lack of established tissue biomarkers that can distinguish between parathyroid cancer (definite carcinoma) and atypical adenoma (or suspicious carcinoma) (22). It has been suggested that this malignancy can be suspected in the presence of particularly high preoperative serum PTH and calcium levels and that extensive surgery is not always necessary (23). A more definitive determination may only be possible with the discovery of the CDC73 pathogenic variant. Therefore, the exploration of gene mutations and the study of recurrence mechanisms will continue to be a research direction for parathyroid cancer.

Our study has some limitations. The database we selected only includes articles under the word mesh in PubMed’s database and does not include articles from other databases. However, taking only mesh words is relatively less in number but more precise in scope. These are common limitations in bibliometrics studies. In addition, artificial intelligence produced the LDA topics and their relationships in this study, presenting machine-driven comprehension. A more in-depth examination of these themes in the future could yield more accurate results and provide a greater depth of understanding.

## Conclusions

There are huge unmet research needs for parathyroid carcinoma. Improving the diagnosis rates of parathyroid cancer by clinicians and establishing new and reliable molecular pathological markers and new image localization techniques will continue to be the focus of future research.

## Data Availability Statement

The raw data supporting the conclusions of this article will be made available by the authors, without undue reservation.

## Author Contributions

YF, SC: conceptualization. CF, CT methodology. CF, LH, HC: formal analysis. CF, CT: investigation. CF and CT: writing—original draft preparation. YF, SC: writing—review, and editing. YF, SC supervision. All authors contributed to the article and approved the submitted version.

## Funding

This work is supported by the Hunan Provincial Natural Science Foundation of China [grant no. 2021JJ30929] and the National Natural Science Foundation of China [grant nos. 81802620].

## Conflict of Interest

The authors declare that the research was conducted in the absence of any commercial or financial relationships that could be construed as a potential conflict of interest.

## Publisher’s Note

All claims expressed in this article are solely those of the authors and do not necessarily represent those of their affiliated organizations, or those of the publisher, the editors and the reviewers. Any product that may be evaluated in this article, or claim that may be made by its manufacturer, is not guaranteed or endorsed by the publisher.
