# Supplementary material for: A Bibliometric Analysis of the Landscape of Parathyroid Carcinoma Research Based on the PubMed (2000–2021)
Source: Front Oncol. 2022 Feb 7;12:824201. doi: 10.3389/fonc.2022.824201 (PMC8858798; doi:10.3389/fonc.2022.824201)
Supplement: Supplementary file 1 [file Table_1.docx]

Supplementary Material

**Supplementary Table. Overall 50 topics identified by LDA.**

| No. | LDA Topic |
| --- | --- |
| 1 | Parathyroid Hormone |
| 2 | Primary Hyperparathyroidism |
| 3 | Cinacalcet for the Treatment of Primary Hyperparathyroidism |
| 4 | Sestamibi Parathyroid Scan |
| 5 | Follow-up Studies |
| 6 | Surgical Innovation |
| 7 | Fine Needle Aspiration Cytology of Parathyroid Lesions |
| 8 | Secondary Hyperparathyroidism |
| 9 | Surgical Treatment |
| 10 | Unilateral and Bilateral Neck Exploration |
| 11 | Multiple Endocrine Neoplasia Type 1 |
| 12 | Minimally Invasive Parathyroidectomy |
| 13 | Comparison of Preoperative Assessment |
| 14 | Single Gland and Multigland Parathyroid Disease in PHP |
| 15 | Retrospective Surgical Treatment Studies |
| 16 | Clinical and Histological Diagnostic Features |
| 17 | Calcium-Sensing Receptor and Vitamin D Receptor |
| 18 | MIBI Parathyroid Scintigraphy |
| 19 | Parathyroid Gland Pathology |
| 20 | Metastases to the Parathyroid Glands |
| 21 | Data Analysis |
| 22 | PTH Levels |
| 23 | Parathyroid Adenoma |
| 24 | Imaging Techniques for Parathyroid Localization |
| 25 | Biochemical Diagnosis of Primary Hyperparathyroidism |
| 26 | Rare Case Reports |
| 27 | Diagnostic Management Strategies |
| 28 | Causes of Primary Hyperparathyroidism |
| 29 | Mediastinal Ectopic Parathyroid Adenoma |
| 30 | Severe Hypercalcemia |
| 31 | Gene Expression of Parathyroid Tumors |
| 32 | Parafibromin Immunostainings of Parathyroid Tumors |
| 33 | Radioguided Parathyroidectomy |
| 34 | Dual Phase Parathyroid Scintigraphy |
| 35 | MEN1 Mutations |
| 36 | Parathyroid Disease Symptoms |
| 37 | Evaluation of A Neck Mass |
| 38 | Surgical Treatment of Concomitant Thyroid and Parathyroid Disorders |
| 39 | Localization Accuracy of Different Imaging Tools |
| 40 | Hyperparathyroidism Caused by Parathyroid Adenoma |
| 41 | Research Aims and Objectives |
| 42 | Parathyroid Hyperplasia |
| 43 | Serum Calcium Levels |
| 44 | Identification of Lymph Nodes and Parathyroids |
| 45 | Repetitive Surgery |
| 46 | Brown Tumors and Parathyroid Tumors |
| 47 | Treatment Outcome |
| 48 | Case Studies of Parathyroid Adenoma |
| 49 | Intraoperative Parathyroid Monitoring |
| 50 | Risks for Hyperparathyroidism |
